# RPI-Bind: a structure-based method for accurate identification of RNA-protein binding sites

**DOI:** 10.1038/s41598-017-00795-4

**Published:** 2017-04-04

**Authors:** Jiesi Luo, Liang Liu, Suresh Venkateswaran, Qianqian Song, Xiaobo Zhou

**Affiliations:** Center for Bioinformatics and Systems Biology and Department of Radiology, Wake Forest School of Medicine, Winston-Salem, NC 27157 USA

## Abstract

RNA and protein interactions play crucial roles in multiple biological processes, while these interactions are significantly influenced by the structures and sequences of protein and RNA molecules. In this study, we first performed an analysis of RNA-protein interacting complexes, and identified interface properties of sequences and structures, which reveal the diverse nature of the binding sites. With the observations, we built a three-step prediction model, namely RPI-Bind, for the identification of RNA-protein binding regions using the sequences and structures of both proteins and RNAs. The three steps include 1) the prediction of RNA binding regions on protein, 2) the prediction of protein binding regions on RNA, and 3) the prediction of interacting regions on both RNA and protein simultaneously, with the results from steps 1) and 2). Compared with existing methods, most of which employ only sequences, our model significantly improves the prediction accuracy at each of the three steps. Especially, our model outperforms the catRAPID by >20% at the 3^rd^ step. All of these results indicate the importance of structures in RNA-protein interactions, and suggest that the RPI-Bind model is a powerful theoretical framework for studying RNA-protein interactions.

## Introduction

RNA-protein interactions are critical at many regulatory steps of gene expression and stages of organismal development^1–5^. Their interactions may vary according to sequences and structures, and consequently perform distinct functions. For example, tRNAs are bound to aminoacyl-tRNA synthetases for the translation during protein synthesis^6^, and nascent RNA coordinates the transition of RNA polymerase (RNAP) II to regulate their own transcription^7^. A large class of long noncoding RNAs (lncRNAs) can bind and modulate the activity of chromatin proteins, and play roles in chromatin modifications^8–13^. In this process, lncRNAs, e.g. the *Xist*, with specific structures can localize chromatin-remodeling complex, such as DNMT3a and possibly also EZH2, to specific target regions whereby stable epigenetic gene silencing can be initiated^14–16^, or act as a scaffold, e.g. the Hotair, to bind more than two proteins with their modules and direct them to target loci^17,18^. It is now apparently observed that many lncRNAs are the key regulators of transcriptional and translational output^1,19–21^, in addition to other genetic and epigenetic regulators^22–29^.

The development of high-throughput experimental methods, such as CLIP-seq and RIP-seq, has greatly advanced the genome-wide studies of RNA-protein interactions^30,31^. Multiple works have been reported to map the full spectrum of RNA interactions of individual RNAs and proteins. For instance, the genome-wide binding of *Xist* and its silencing partners have been profiled^32–34^. Very recently, Hendrickson *et al*. performed experiments with fRIP-Seq to detect widespread binding of mRNA and lncRNA with 24 proteins^35^. However, these experimental methods are always expensive, time-consuming and labor-intensive.

It is necessary to develop computational approaches to efficiently investigate RNA-protein interactions. A few methods have been reported for three purposes: 1) the investigation of associations between proteins and RNAs, such as RPI-Pred^36^, RPIseq^37^, and lncPro^38^; 2) the prediction of binding sites on either RNAs or proteins, such as the sequenced-based methods: BindN^39^, RNABindR^40^, RNAProB^41^, PPRint^42^, RNApin^43^, PRINTR^44^, RISP^45^, PiRaNhA^46^, BindN+^47^, NAPS^48^, PRBR^49^, SRCPred^50^, Predict_RBP^51^, RNABindRPlus^52^ and RBRIdent^53^; the structure-based methods: KYG^54^, RsiteDB^55^, PRIP^56^, OPRA^57^, DRNA^58^, PRNA^59^, aaRNA^60^, RBRDetector^61^ and RBscore^62^; and 3) the residue-nucleotide contacts prediction, such as catRAPID^63^. The catRAPID method is the only available method and different from those are specially designed to determine residue-nucleotide interactions of different known DNA-binding domain families^64,65^. The latter cannot be applied to study RNA-protein binding interactions, since RNA is more flexible than DNA and has more complicated structures.

Most of the existing methods only employ the sequences of proteins or RNAs, although some, such as catRAPID, implement other information like van der Waals contacts, hydrogen bonds, electrostatic interactions and stacking interactions across the protein-RNA interfaces. However, it is well known that, in many cases, the structures of molecules, including both proteins and coding/non-coding RNAs, dictates their functions^10,66–72^. As an example, the RPI-Pred, using high-order three-dimensional protein and RNA structures, significantly improves the accuracy in contrast to others using only sequences.

**Figure 1:**
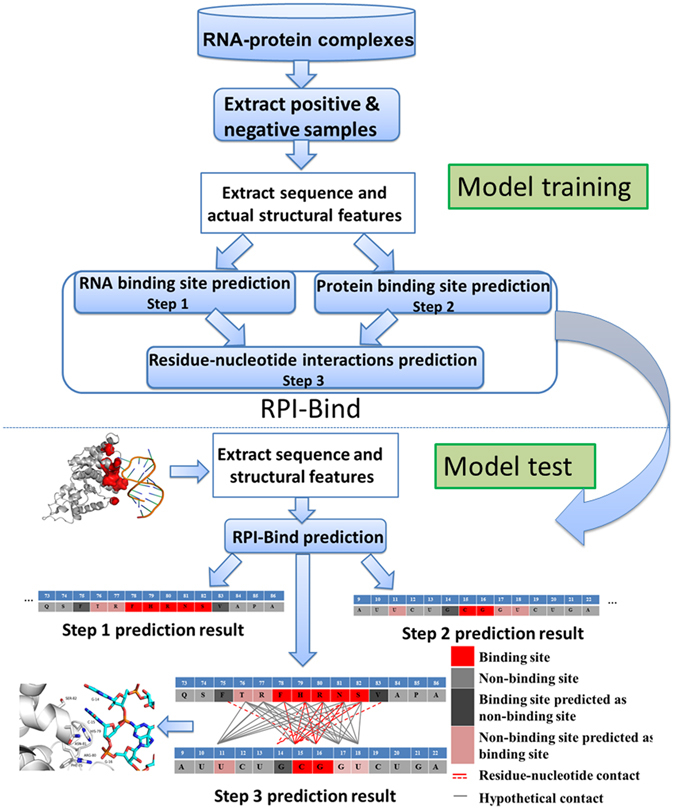
The step-wise work flow of the RPI-Bind prediction method. The whole work flow consists of two steps: training classification models and the applications. The model training process includes various processes, such as construction of the training dataset, feature extraction from sequences and structures in the training data set and development of ‘RPI-Bind’ method, consisting of three models. The developed models were then applied to solve three problems, including 1) the prediction of RNA binding regions on protein, 2) the prediction of protein binding regions on RNA, and 3) the prediction of interacting regions onboth RNA and protein simultaneously.

In this work, we implemented both sequences and structures of RNAs and proteins for the study of RNA-protein binding sites. To represent structures, we used the protein local conformations (PLCs), named protein blocks (PBs)^73^, and 12 classes of RNA local conformations (RLCs) from ‘BEAR’ encoding^74^, respectively. Both of them can give more detailed descriptions of RNA and protein structures than other representations^73,74^. We firstly illustrated the preferring properties of PLCs/RLCs for RNA-protein interactions. For instance, the α-helix and β-sheet PLCs, and the stem RLCs are^64^ preferred to exist at protein-RNA interfaces. We then developed a three-step RPI-Bind method to identify the binding sites on a given pair of protein and RNA. The three steps include 1) the prediction of RNA binding regions on protein, 2) the prediction of protein binding regions on RNA, and 3) the prediction of interacting regions on both RNA and protein simultaneously (Fig. 1). We showed that in the 1^st^ and 2^nd^ steps, the inclusion of structures of RNAs and proteins can increase the prediction accuracy of RNA binding regions on protein, and RNAs, respectively. More importantly, at the 3^rd^ step, the inclusion of structures and the predicted results from the first two steps can lead to an accuracy of ~86.9%, significantly higher than the catRAPID (~62%)^63^. We also applied the RPI-Bind method to identify the interacting regions of lncRNA *Xist* and transcription factor YY1, as well as other 20 proteins^35^. The results show great agreements between our predictions and experimental measurements, indicating the RPI-Bind is a powerful theoretical framework for the study of RNA-protein interactions.

## Results

### Statistical analysis of PLCs and RLCs at RNA-protein interfaces

We extracted 172 non-redundant RNA-protein interacting pairs (Supplemental Table S1) by filtering the pairs from the Nucleic Acid Database (NDB)^75^ and the Protein Data Bank (PDB)^76^. We then constructed a database consisting of 28,780 nucleotide-residue contacts, consisting of 9,077 RNA binding sites (on proteins) and 5,692 protein binding sites (on RNAs), respectively. Meanwhile, 9,801 RNA non-binding sites and 3,078 protein non-binding sites were also collected for further analyses. The protein and RNA structures were analyzed with the PDB-2-PB database^77^ and the ‘BEAR’ approach^74^ for the PLC and RLC representations, respectively (Supplemental Tables S2 and S3).

**Figure 2:**
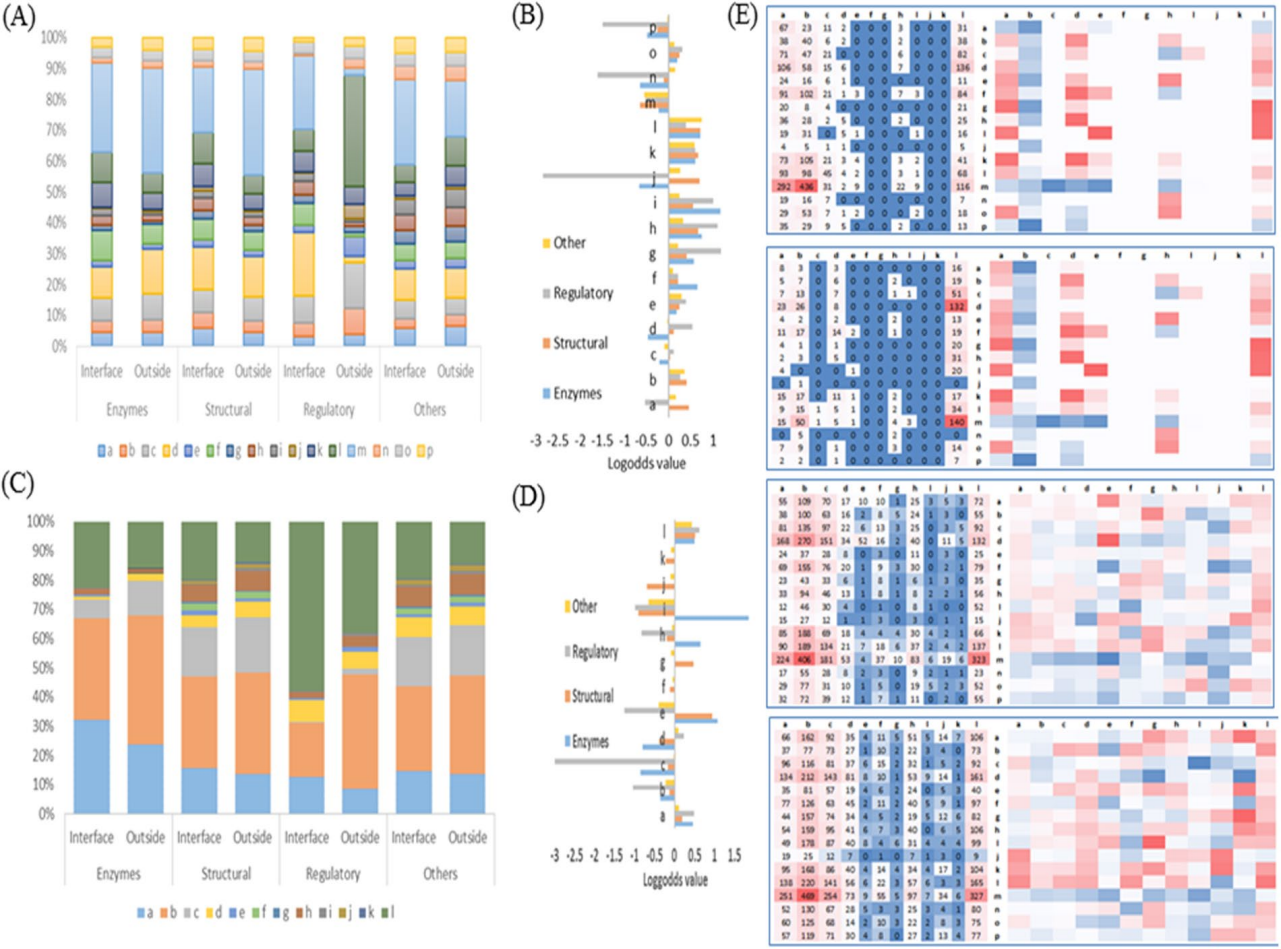
Statistical analysis of protein local conformations (PLCs) and RNA local conformations (RLCs) at and outside the interface for the four types of protein functional classes. (**A**) and (**C**) show the composition percentages of PLCs and RLCs at and outside the interfaces. The corresponding log-adds ratio to represent over and less at and outside the interfaces for PLCs and RLCs are given in (**B**) and (**D**). The mutual interaction propensity matrices between PLCs and RLCs are shown in (**E**). The left side values in (**E**) represent the total number of contacts between PLCs and RLCs and right side boxes represent their corresponding log-odds values. The four classes are shown from top to bottom, are enzymes, structural, regulatory and ‘other’, respectively.

We analyzed the PLCs/RLCs compositions, preferences and their mutual interaction propensities at the interfaces of four classes of non-redundant protein-RNA complexes, including enzymes, structural, regulatory and ‘others’, with each contains 40, 48, 34 and 50 protein-RNA pairs, respectively. By comparing the interface and outside PLCs among the four classes, the most populated PLC at the interface is the d type PLC, representing β-sheet, for the regulatory class (Fig. 2A,B and Supplemental Table S2). Other PLCs show the similar distributions for these four classes of protein-RNA complexes. The m and d type PLCs that represent α-helix and β-sheet are also overpopulated in all four classes, followed by the N-terminal α-helix and β-sheet PLCs (l, f, k, c, a and b types). By contrast, the C-terminal α-helix, β-sheet and coil PLCs (e, f, n, o, p, g, h, l, and j types) show unfavorable at the binding interfaces. Overall, the high preferences of l, k, h and g types were observed. All PLCs do not show much different preferences among the four classes of protein-RNA complexes, except that j, p, and n types have the lowest preferences in the regulatory class (Fig. 2A,B and Supplemental Table S2). The overall local structure description allows us to understand the protein-RNA binding nature in terms of structural fragments.

For RNAs, the major difference lies in the c type RLC, representing stem branch with less number in regulatory class (Fig. 2C and D). The b and l type RLCs, representing stem and unknown regions respectively are highly overpopulated in all four classes of protein-RNA complexes. Followed by stem branch, left internal loop, bulge left, left internal loop branch and bulge left branch (c, d, e, f and g types) are also populated. The Right internal loop, Bulge right, Right internal loop branch, and Bulge right branch (h, i, j and k types) show few or no contacts throughout all four classes (Supplemental Table S3).

The mutual interaction propensity (MIP) matrices of PLCs and RLCs were analyzed for quantitative evaluation of protein/RNA structure preferences. According to the total number of occurrences, the pair of α-helix and stem (m-b pair) appears the most at the interface for the enzymes, structural and ‘others’ classes (Fig. 2E). While in the regulatory class, α-helix and unknown pair (m-l pair) is highly preferred. Among all PLCs and RLCs, α-helix and β-strand PLCs (m and d types), and loop, stem and unknown RLCs (a, b, and l types) have the most contacts. Overall, the enzymes and regulatory classes share similar interaction propensities: the RLCs, including loop, left internal loop, right internal loop and unknown (a, d, h, and l types), show high propensities for interacting. In both structural and ‘others’ classes, many pairs have high interaction propensities, but the highest propensities were observed for β-strand – bulge left pair (d-e pair) and coils–bulge right branch pair (g-k pair), respectively (Fig. 2E).

**Figure 3:**
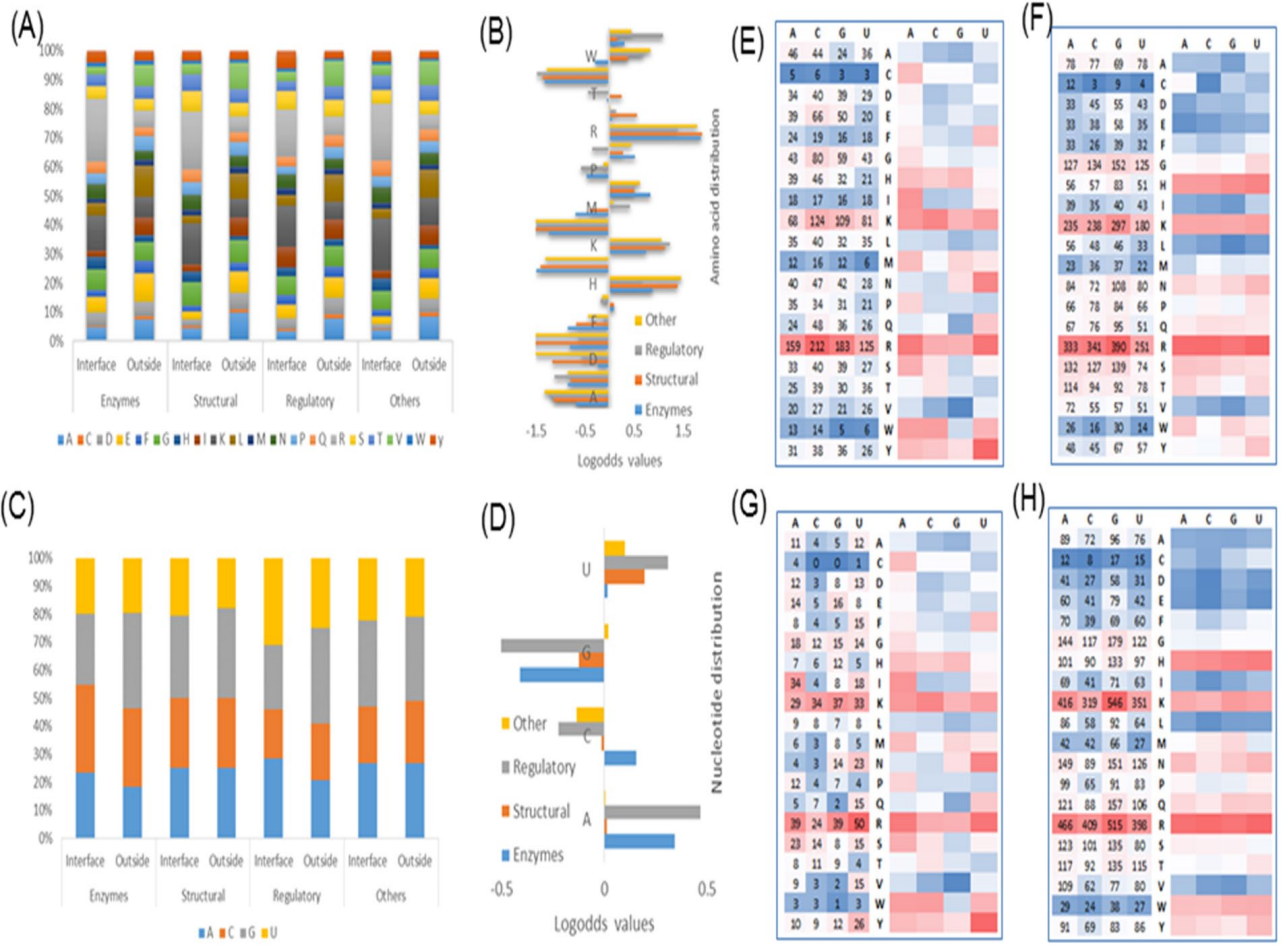
The occurrences of amino acid and nucleotide sequences at and outside the interface for all types of protein functional classes (enzymes, structural, regulatory, and other). The occurrences of amino acid and nucleotide at the interface and outside are shown in (**A**) and (**C**), respectively. In (**B**) and (**D**), the log-odds ratio of amino acid and nucleotide shows the over and less populated amino acid and nucleotides at and outside the interfaces. Further, the mutual interaction propensities (log-odds value) between amino acids and nucleotides are given for all four classes in (**E–H**), respectively. In each figure, the values on the left side represent the total number of contacts between residue and nucleotide and right side boxes represent their corresponding log-odds values.

In addition to the PLCs and RLCs, we also analyzed the compositions, preferences and interaction propensities of amino acids and nucleotides from the four classes of protein-RNA complexes (Fig. 3). The positively charged residue, arginine and lysine, and the single aromatic residues, phenylalanine and tyrosine, play key roles in the RNA binding sites, consistent with the previous studies^78,79^. RNA-binding proteins achieve RNA-binding affinity through favorable charge-charge interactions between positively charged Arg and Lys residues and the negatively charged RNA phosphate. The observed high propensity of single aromatic residues reflects the frequent stacking interactions between aromatic side chains and nucleic acid bases in a number of protein-RNA complexes. In contrast to the observation of enormous variances in individual residue at the interface, few nucleotide variations were observed among protein binding sites. This could be the reason why very few methods are currently available to predict the binding sites of proteins on RNAs.

### PLCs enable highly accurate prediction of RNA binding regions on proteins

We then start to build the first model (1^st^ step) to predict RNA binding regions on proteins for the three-step RPI-Bind (RNA-protein binding region predictor) method (Fig. 1).

We developed a Random Forest (RF)-based machine learning method for the prediction of RNA binding sites on proteins, by firstly using the features of sequence mutual interaction propensities, physicochemical characteristics, hydrophobic index, relative accessible surface area, conservation score and side-chain environment, respectively. These features were chosen because they had been shown to outperform other RNA binding residue prediction methods^59^. Within the constructed database of 9,077 RNA binding sites and 9,801 RNA non-binding sites, we used a standard five-fold cross validation procedure to estimate the performance. The Sensitivity (SN), Specificity (SP), Accuracy (ACC), and Matthew’s Correlation Coefficient (MCC) were 66.8%, 74.8%, 71.3% and 0.425, respectively.

**Figure 4:**
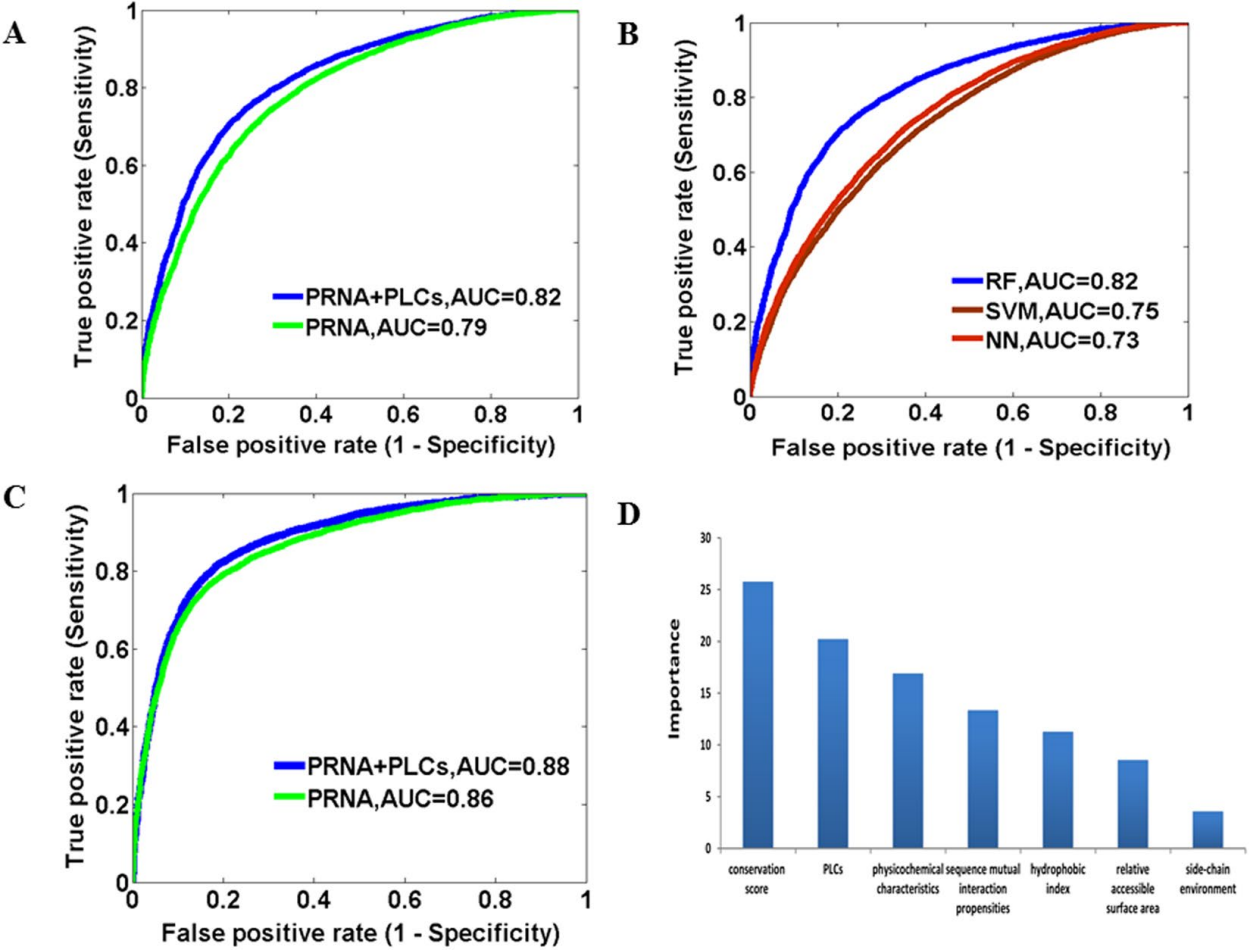
The performance of RNA binding site prediction. (**A**) Comparison of ROC curves for binding site prediction using different features on our constructed database. (**B**) Comparison of ROC curves for binding site prediction using different classifiers. (**C**) Comparison of ROC curves for binding site prediction on an independent dataset. (**D**) The importance and individual contribution ratio of each feature type.

We then combined the structure local conformations features of protein and RNA structures (PLCs and RLCs), because it could be better to predict binding residues. A five-residue sliding window method was used, as it outperforms other (3, 7, or 9) size windows. If the center residue or nucleotide in each window was detected to physically interact with each other, then the window was treated as a positive sample, otherwise negative. The performance could be improved to 71.1%, 77.7%, 74.8% and 0.489, respectively. The ROC curves were also adopted to show the prediction accuracy (Fig. 4A).

We also employed other machine learning methods for the model development, including Support Vector Machine (SVM) and Neural Network (NN). The result shows that the RF-based model achieves the best prediction (Fig. 4B). Therefore we selected the RF for the following analyses.

To evaluate the performance of our model and the contribution of structures in RNA-protein interactions, we applied the developed model to an independent dataset from the PRNA method^59^. It should be noted that the independent dataset and our non-redundant dataset have partially overlaps. There are 26 common protein chains in the two datasets. So the redundant protein chains were removed from independent dataset and the final dataset contains 3,584 (10.3%) interacting residues and 31,284 (89.7%) non-interacting residues. Our model can achieve predictions with ACC of 81.3%, PPV of 31.9%, NPV of 97.5% and the Area Under ROC curves (AUC) of 0.88 (Fig. 4C), compared to the PRNA with the ACC, PPV, NPV and AUC as 78.5%, 27.3%, 96.2% and 0.86. Furthermore, we test the 1^st^ step model on a recent data set, RBscore_R130^62^. The common protein chains to our non-redundant dataset and independent dataset were removed from RBscore_R130. Our model exhibits high prediction accuracy on this set with 0.8305 AUC, compared with 0.8063 AUC for BindN+^47^, 0.82 for RNABindRPlus^52^, 0.8383 for aaRNA^60^, 0.7501 for PPRint^80^ and 0.7479 for KYG^54^.

We also used the permutation importance analysis to evaluate the contribution of individual feature type in predictions (Fig. 4D). The importance score of our local conformations feature is 20, which is higher than that of other features except the conservation score. These results indicate that structures (local conformation features) make great contribute to the prediction of RNA binding sites and could improve the prediction performance.

**Figure 5:**
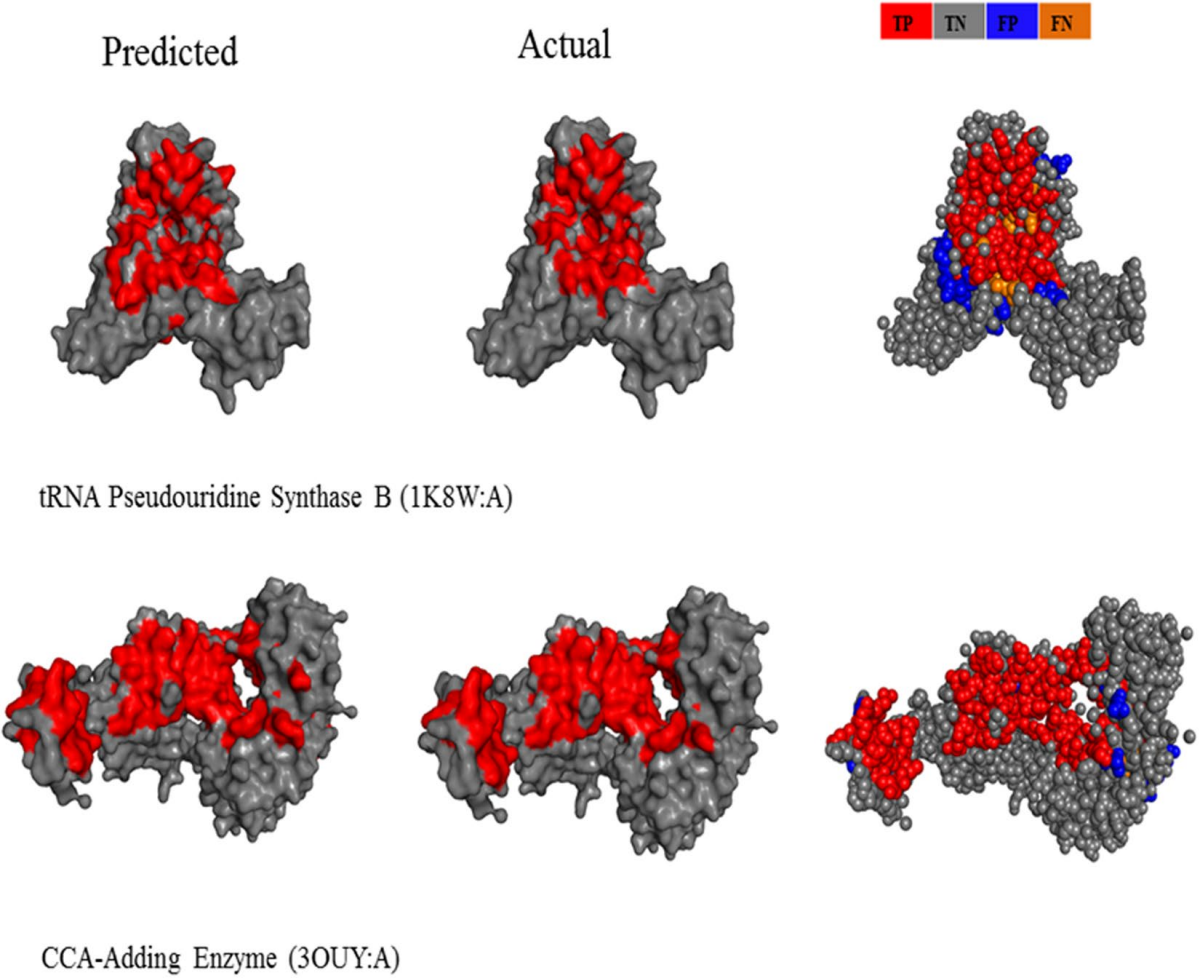
Examples of predicted RNA-protein interacting complexes. The two examples are tRNA Pseudouridine Synthase B and CCA-Adding Enzyme Predicted RNA binding sites are shown in red and predicted non-binding sites in gray (left panels). Actual RNA binding sites in red and actual non-binding sites in gray (middle panels). The performance of prediction for individual residues, with true positives (TP) shown in red, false positives (FP) in blue, false negatives (FN) in orange, and true negatives (TNs) in gray (right panels). Thus, red + orange residues correspond to the actual binding residues; red + blue residues correspond to the predicted binding residues. All structure diagrams were generated using PyMol (http://www.pymol.org).

The Fig. 5 shows examples in which the prediction method was tested with two RNA binding proteins, including CCA-Adding Enzyme (PDB 3OUY:A)^81^, and tRNA Pseudouridine Synthase B (PDB 1K8W:A). The CCA-adding enzymes are nucleotidyltransferases that catalyze the posttranscriptional addition of the nucleotide sequence CCA onto the 3′ terminus of immature tRNA without using a nucleic acid template, and tRNA Pseudouridine Synthase B catalyzes the isomerization of specific uridines in cellular RNAs to pseudouridines and may function as RNA chaperones. For the former, our method correctly identified 89 of 97 actual interface residues, and for the latter, we predicted 63 out of 83 actual interface residues. The prediction accuracy for each RNA-protein complex is shown in the Supplemental Table S4. These results prove the predictive ability of our model.

### RLCs enable highly accurate prediction of protein binding regions on RNAs

We also built a RF-based machine learning method for the prediction of protein binding sites on RNAs, firstly using three compositional features (the 2^nd^ step of our approach, Fig. 1), including mono-, di- and tri-nucleotide composition profile^43^. Similarly, within the constructed database of 5,692 protein binding sites and 3,078 protein non-binding sites, we used a standard five-fold cross validation procedure to estimate the performance, that is, SN, SP, ACC and MCC were 72.6%, 54.1%, 63.5% and 0.271, respectively.

**Figure 6:**
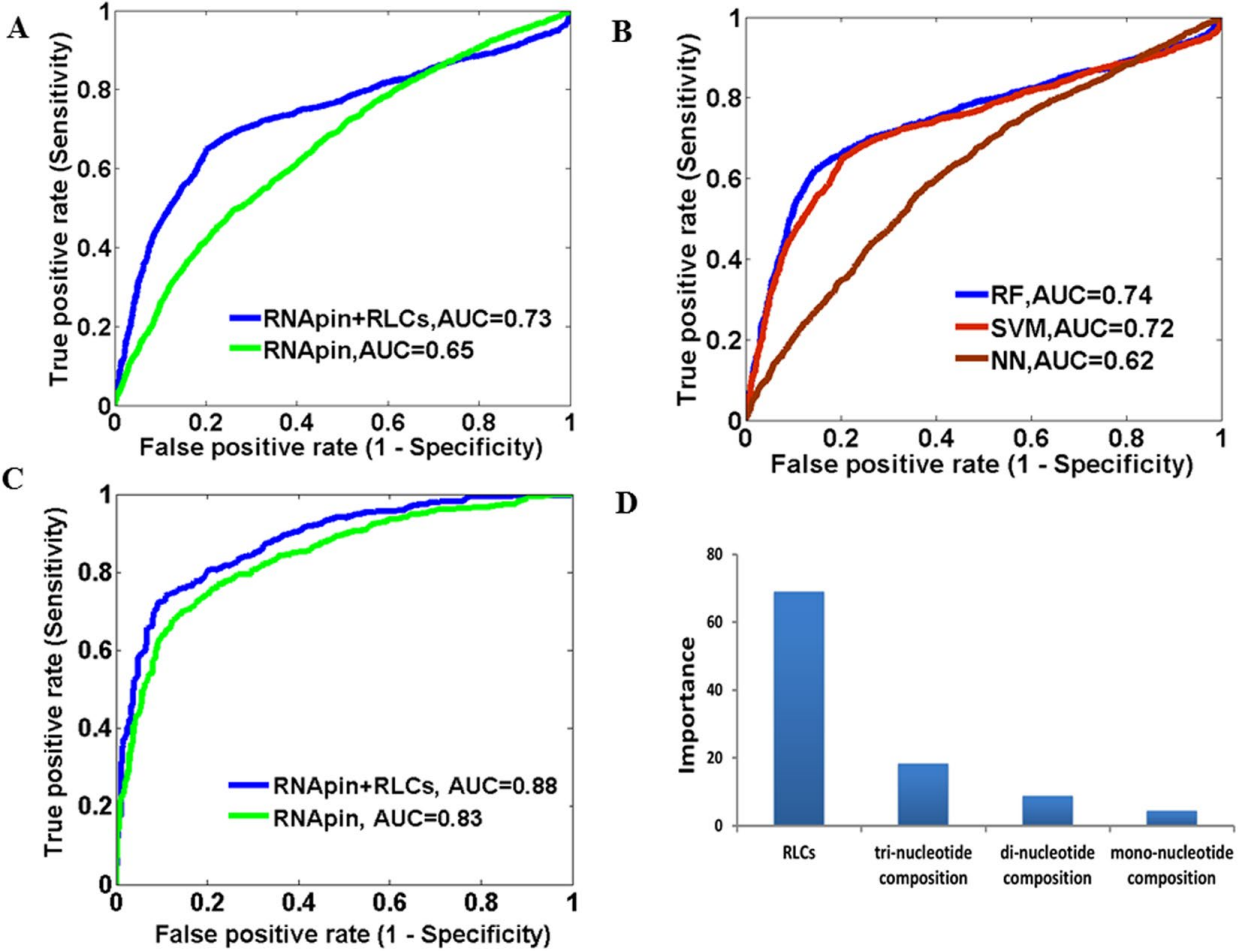
The performance of protein binding site prediction. (**A**) Comparison of ROC curves for binding site prediction using different features on our constructed database. (**B**) Comparison of ROC curves for binding site prediction using different classifiers. (**C**) Comparison of ROC curves for binding site prediction on an independent dataset. (**D**) The importance and individual contribution ratio of each feature type.

When additional structural features were used, the prediction could reach 77.4%, 65.2%, 71.4% and 0.4 for SN, SP, ACC and MCC, respectively (Fig. 6A). Other machine learning methods, such as SVM, also give similar performances (Fig. 6B). Therefore, the five-residue window and the RF were selected for the model building and following analyses. We then applied our method to a ‘RNA208’ dataset to compare the performance with the RNApin^43^, one of the two methods currently available to predict protein interacting nucleotides in RNAs and having better performances^43,82^. We also removed 23 overlapped RNA chains from the ‘RNA208’ dataset. Our method outperformed the RNApin (77.6% ACC, 55.7% PPV, 94.6% NPV and 0.83 AUC) with ACC of 81.9%, PPV of 59.5%, NPV of 95.0% and AUC of 0.88 on the new dataset (Fig. 6C). More importantly, the importance score of RNA local conformations feature is as high as 70, by applying the permutation importance analysis (Fig. 6D), implying that the structures contain more important information than the sequence only.

**Figure 7:**
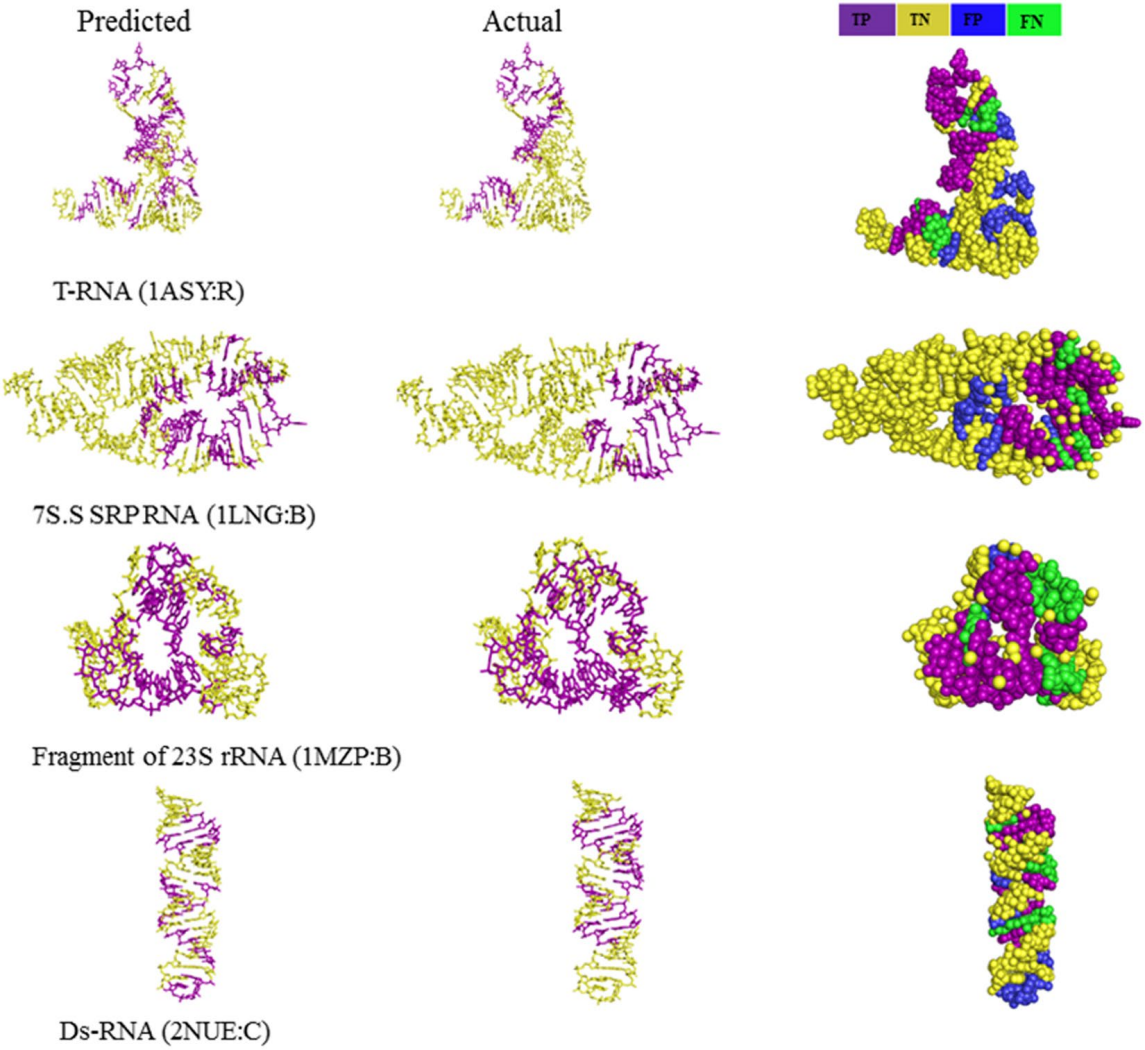
Examples of predicted RNA-protein interacting complexes. Examples of prediction results for four different RNA are shown from top to bottom, are T-RNA, 7S.S SRP RNA, Fragment of 23S rRNA and dsRNA, respectively. Predicted protein binding sites are shown in purple and predicted non-binding sites in yellow (left panels). Actual RNA binding sites in purple and actual non-binding sites in yellow (middle panels). The performance of prediction for individual nucleotides, with true positives (TP) shown in purple, false positives (FP) in blue, false negatives (FN) in green, and true negatives (TNs) in yellow (right panels). Thus, purple + green nucleotides correspond to the actual binding nucleotides; purple + blue nucleotides correspond to the predicted binding nucleotides. All structure diagrams were generated using PyMol (http://www.pymol.org).

The Fig. 7 shows examples of our predictions. Our model correctly identified 19 of 24 actual interface nucleotides for the 7S.S SRP RNA^83^. The SRP (signal recognition particle) is a ribonucleoprotein, which associates with ribosomes to mediate co-translational targeting of membrane and secretory proteins to biological membranes. The S domain of SRP interacts with 7S.S RNA for SRP assembly in Eukarya and Archaea. Our model also successfully predicted 18 of 25 actual interface nucleotides for T-RNA, 20 of 27 for fragment of 23S rRNA and 12 of 20 for dsRNA, respectively. These results indicate that our model is reliable for identifying protein binding sites on RNAs (Supplemental Table S4).

### PLCs and RLCs enable residue-nucleotide interaction prediction at RNA-protein interface

The successful prediction of RNA binding sites on proteins and protein binding sites on RNAs in the above two steps of RPI-Bind indicates the importance of structures in the determination of RNA-protein interactions. We further used the structure and sequence information to build the 3^rd^ model of RPI-Bind for the identification of residue-nucleotide interactions at the protein-RNA interfaces, that is, interacted residues in the protein chains and nucleotides in the RNA chains. There are a total of 28,780 residue-nucleotide contacts in our database, here, 70% of them were randomly selected to construct the training set, and the remaining was put into the test set to evaluate the performance of our final model. Each residue-nucleotide interaction was divided into fragments by moving a window of 5 successive residues or nucleotides along the proteins and RNAs. Each window was encoded by structure and sequence features used in the 1^st^ and 2^nd^ steps. We then trained our RPI-Bind model with the combinational features by employing the RF classifier. Our model can achieve predictions with SN of 70.0%, SP of 89.2%, ACC of 79.5%, and MCC of 0.60, evaluated by five-fold cross validation. Then, we further evaluate the practical prediction ability of our model on test set. The SN, SP, ACC and MCC are 73.4%, 83.8%, 79.7% and 0.57, respectively.

Furthermore, we can take the advantage of our predictions from the RNA binding sites on proteins (1^st^ step, Fig. 1) and the protein binding sites on RNAs (2^nd^ step, Fig. 1). We selected the successfully predicted 4,738 (out of 6,334, from step 1) interacting residues and 2,608 (out of 3,984, from step 2) interacting nucleotides, which form 11,211 true residue-nucleotide contacts from our training set. We firstly used five-fold cross validation to estimate the performance of new training set. The average SN, SP, ACC and MCC are 84.0%, 90.0%, 86.9% and 0.76, respectively. The performance of the new training set was also evaluated on test set, the SN, SP, ACC and MCC are 82.4%, 95.1%, 91.4% and 0.79, respectively. These results show that the inclusion of structures and the predicted results from the first two steps significantly increase the prediction accuracy of residue-nucleotide contacts. Our method significantly outperforms the catRAPID method, the prediction accuracy of which is close to 62%.

**Figure 8:**
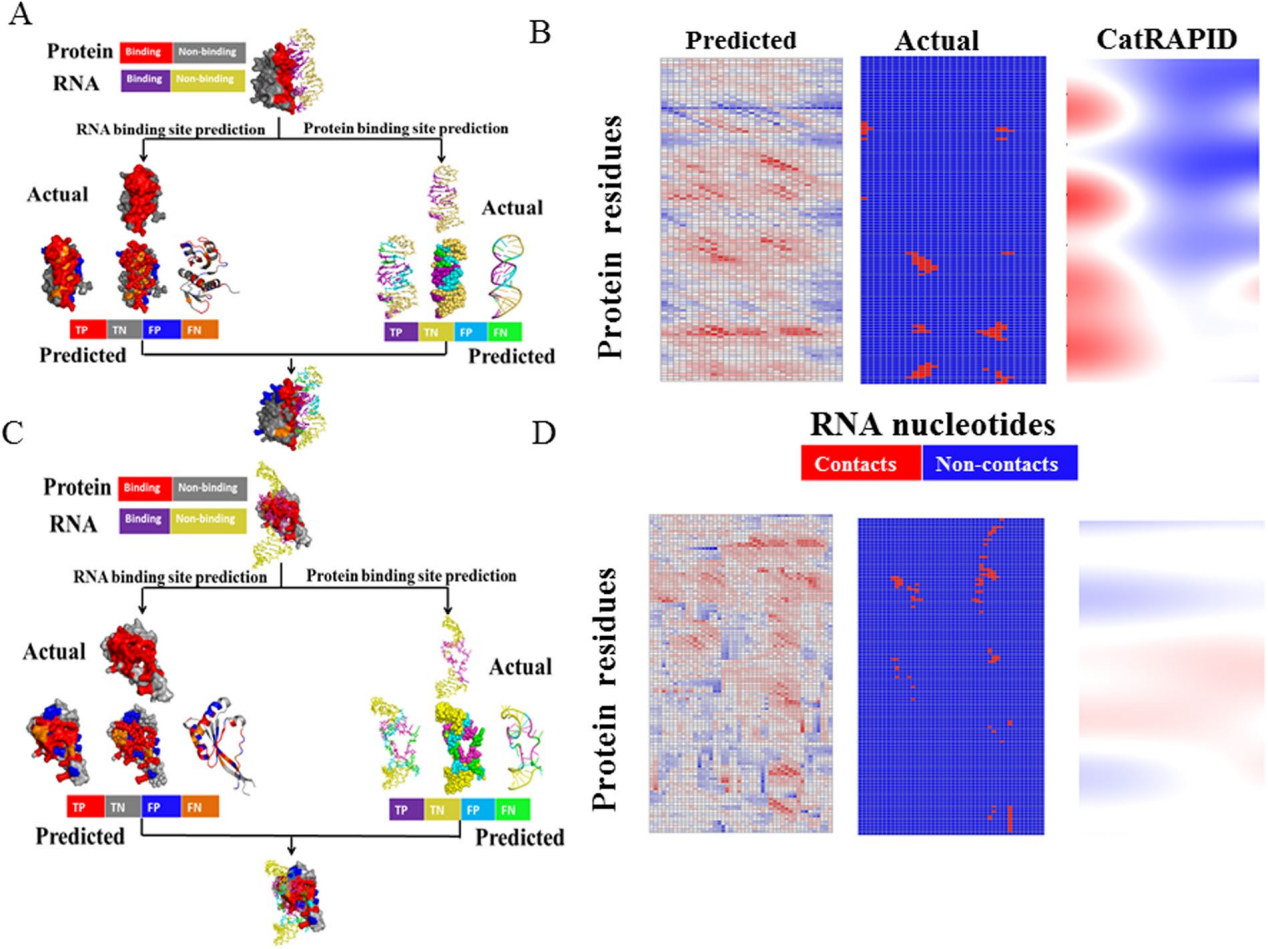
Two example of protein-RNA complexes (PDB id: 1I6U and 3IAB). (**A**) and (**C**) Protein and RNA binding sites prediction results of 1I6U and 3IAB, respectively. The results are mapped onto the original structure where different prediction catalogs are represented by different colors; (**B**) and (**D**) Comparison of residue-nucleotide contacts prediction results by our 3^rd^ step model and the catRAPID method (http://service.tartaglialab.com/page/catrapid_group).

Test of two individual RNA-protein interacting complexes (PDB id: 1I6U and 3IAB) also indicates that our model is reliable for residue and nucleotide interaction prediction (Fig. 8A and C). Our 1^st^ step model of RPI-Bind correctly predicted 28 of 40 and 34 of 46 RNA interacting residues for the 1I6U and 3IAB, respectively. Then the 2^nd^ step model correctly predicted 11 of 14 and 10 of 16 protein interacting nucleotides, respectively. For the residue-nucleotide contacts prediction, our 3^rd^ step model correctly predicted all actual interactions. On the other hand, 2% of non-contacts was wrongly predicted as contacts (false positives), if the lowest score of actual interactions was set as a cutoff (Fig. 8B and D). In contrast, the catRAPID failed to identify the actual residue-nucleotide contacts and the majority of residues and nucleotides in the protein chain and RNA chain were predicted as binding sites. All of these results indicate the outperformance of our model in RNA-protein binding prediction, and the crucial roles of RNA and protein structures in their interactions.

### Predicting protein associations with lncRNAs

LncRNAs play essential roles in a variety of biological process^84^, and are implicated in serial steps of cancer development^20^. LncRNAs mainly perform the biological functions by interacting with different proteins^13^. Our previous RPI-Pred method^36^ can accurately predict protein-lncRNA interaction pairs or identify the binding partners of a given protein or RNA. On the basis of RPI-Pred, our three-step RPI-Bind model can further predict the interaction region between protein and lncRNA, and therefore can be useful for the study of lncRNA functions.

The first application of our model is to investigate the interaction between lncRNA *Xist* and a transcription factor YY1. Their specific contacts are required to load *Xist* onto X chromosome^85^. Due to the lack of solved structures, we applied PB-kPRED^86^ and RNAfold^87^ for the local conformation prediction for YY1 and *Xist*, respectively. As well, the *Xist* is very large, so in order to obtain better lncRNA structures, functionally important segments, A, F, B, C, and E, were selected, and analyzed separately. Our 1^st^ step model predicted three regions of YY1 (the sequence from 60–80, 183–210 and 303–320) are interacted with *Xist*, while the 2^nd^ step model predicted the B, C and E segments of *Xist* are more likely to associate with YY1. With our 3^rd^ step model, we successfully identified that the sequence from 60–80, 183–210 and 303–320 of YY1, and the B and C segments of the *Xist* are really contacted (Supplemental Fig. S1), in consistence with experimental evidence^85^.

The second application of our model is to identify the binding regions between *Xist* and other 20 chromatin-associated proteins, such as EZH2 and CHD4, which have been recently determined with the fCLiP-seq technique^35^. Our goal is to predict potential regions that associate with a number of proteins involved in epigenetic regulation on the *Xist*. The results shown by the heat-map is very intuitive and straight-forward (Supplemental Fig. S2). Our model predicts that *Xist* segment E (6990–9467 nt) binds strongly to EZH2, CHD4, SUZ12 and HNRNPU, in agreement with experimental evidence. EZH2 also shows high interaction propensity to segment D (5550–5730). Neither segment A nor F is predicted to be in contact with any of the 20 chromatin-associated proteins except SUZ12. On the other hand, no or low interactions were found between four proteins (HDAC1, CHD1, HUR and CBX3) and any functional segments of *Xist*. For those *Xist* non-binding proteins (RBBP5, CBP, CHD7, DNMT1, ADAR, CTCF, PCAF, NUP98, WDR5, LSD1, IMP1), our method correctly predicted that they do not bind to any functional segment of *Xist*. Our model also predicts some unknown regions of *Xist*, such as *Xist* 3′terminus, showing high propensity to contact most of proteins, which implies that these regions might be new functional segments. In summary, our model provides highly accurate predictions of RNA-protein interactions, in agreement with experimental evidence (Supplemental Fig. S2).

## Discussion

RNA-protein interactions are of importance for the function of RNAs, especially lncRNAs^8–13^. Numerous methods have been proposed for the identification of these interactions; however, two main limitations exist for those methods: 1) most of them are only able to identify the interacting regions of either RNA on protein or protein on RNA^40–43,88^. The model for residue-nucleotide contacts prediction is catRAPID, which unfortunately cannot provide highly accurate identifications. 2) Most of them only use the sequences of proteins or RNAs, but ignore their structures, which, however, are much known to affect their functions and interactions^10,66–72^. In this work, we extracted the general characteristics of protein and RNA binding. To do so, the best approach is to observe the difference between binding and non-binding sites from known structures. However, observation from one single pair of protein-RNA binding structures is always bias, and may not represent the general characteristics of binding/non-binding sites. In contrast, the comparison between the collection of residues including both binding and non-binding ones leads to the detection of these features. Therefore, we first illustrated the structure preferences of binding and non-binding sites at the protein-RNA complexes, and then developed a three-step RPI-Bind model for the prediction of RNA-protein interface by employing the sequence and structure information of RNAs and proteins. Tests show that our RPI-Bind model outperforms other existing models at each of the three levels, including 1) the prediction of RNA binding regions on protein, 2) the prediction of protein binding regions on RNA, and 3) the prediction of interacting regions on both RNA and protein simultaneously. Of note, at the third step, the inclusion of the predicted results from the first two steps can further improve the prediction accuracy, which is significantly better than the catRAPID (86.9% vs 62%). More tests on individual RNA and protein interactions, especially lncRNA-protein interactions, further illustrate the prediction ability of our RPI-Bind model, which, on the other hand, depict the importance of structures in RNA-protein interactions. Our model is freely available at http://ctsb.is.wfubmc.edu/publications/RPI-Bind-Pred.php.

## Materials and Methods

We constructed a non-redundant protein-RNA interacting dataset based on the 1,342 protein-RNA complexes from the Nucleic Acid Database (NDB)^75^ and their corresponding 3D structures in the Protein Data Bank (PDB)^76^. After a series of data processing steps (Supplemental Materials), we obtained 172 non-redundant protein-RNA pairs (Supplementary Table 1). In these protein-RNA pairs, a total of 28,780 residue-nucleotide contacts, consisting of 9,077 RNA binding sites and 5,692 protein binding sites were obtained based on the distances between interacting residues and nucleotides. As well, 9,801 RNA non-binding sites and 3,078 protein non-binding sites were randomly paired to obtain a total of 180,000 pairs as the negative dataset (Supplemental Materials).

The structures of proteins and RNAs were represented by protein local conformations (PLCs) as 16 types of the protein blocks (PBs), and 12 types of RNA local conformations (RLCs) (Supplemental Materials, and Supplemental Tables S2 and S3). We then investigated the PLCs/RLCs compositions, preferences, and their mutual interaction propensities at the interfaces of protein-RNA complexes (Supplemental Materials).

With these observations, we presented a three-step RPI-Bind (RNA-protein binding region predictor) method for the prediction of binding sites on both RNAs and proteins (Fig. 1) as follows:

1. The prediction of RNA binding regions on protein. We extracted sequence and structure features for RNA binding sites to develop the prediction method. The involved features include mutual interaction propensities, physicochemical characteristics, hydrophobic index, relative accessible surface area, conservation score, and side-chain environment, as well as the PLCs descriptors (triplet-log-odds values) (Supplemental Materials). We employed the sliding window approach to decode the amino acid residues of proteins. Whether a residue belongs to the interactions or not is determined by the middle residue. The feature vector representing the residue in the window is encoded by the properties of the included residues. We compared the performance of different window sizes (3, 5, 7 and 9), and the best prediction performance was obtained with a window of 5 residues. We should note that the mutation of protein leads to two different protein sequences, and maybe two different protein structures, at least for local structure. Therefore, the input to the model will be different, which will result in different binding site identification.
2. The prediction of protein binding regions on RNA. The involved features include mono-, di- and tri-nucleotide sequence compositions, and RLCs descriptors (triplet-log-odds values) (Supplemental Materials). We also compared the performance of four window sizes (3, 5, 7 and 9). The five, seven and nine-window size have similar performance, but better than three-window size. We then set the window size as 5.
3. The prediction of interacting regions on both RNA and protein simultaneously. Our goal is to predict all interacting information in a framework. So the involved dataset and features include all used in the 1^st^ step and 2^nd^ step. We presented two models. One uses the combinational features to develop model, and the original dataset consisting of 28,780 residue-nucleotide contacts for training and testing the model. Another also uses the combinational features, but the dataset was constructed with the positive dataset, obtained from the successfully predicted interacting residues and interacting nucleotides at the 1^st^ and 2^nd^ steps, respectively. The original negative dataset consisting of 180,000 pairs was formed by pairing every binding site and neighbor non-binding sites around its interacting partners. We used this strategy to construct negative dataset, because these false contacts are more similar to true contacts. Distinguishing true protein-RNA contacts from these false contacts is more practical.

Machine learning methods, such as the Random Forest (RF), Support Vector Machine (SVM) and Neural Network (NN), were employed for model building at the three steps, and the performance was evaluated with Sensitivity (SN), Specificity (SP), Accuracy (ACC), the Area Under ROC curves (AUC), and Matthew’s Correlation Coefficient (MCC) (Supplemental Materials).

## Electronic supplementary material

Supplemental Materials

Dataset
