# Author Correction: RPI-Bind: a structure-based method for accurate identification of RNA-protein binding sites

**DOI:** 10.1038/s41598-018-23328-z

**Published:** 2018-04-05

**Authors:** Jiesi Luo, Liang Liu, Suresh Venkateswaran, Qianqian Song, Xiaobo Zhou

**Affiliations:** Center for Bioinformatics and Systems Biology and Department of Radiology, Wake Forest School of Medicine, Winston-Salem, NC 27157 USA

Correction to: *Scientific Reports* 10.1038/s41598-017-00795-4, published online 04 April 2017

The Acknowledgements section in this Article is incomplete.

“We would like to acknowledge the members of Center for Bioinformatics and Systems Biology at Wake Forest School of Medicine, especially Dr. Hua Tan, for valuable discussions and advices. This work was supported by National Institutes of Health [1U01CA166886] (to X. Zhou), and partially supported by NSFC No. 61373105. Funding for open access charge: National Institutes of Health [1U01CA166886].”

should read:

“We would like to acknowledge the members of Center for Bioinformatics and Systems Biology at Wake Forest School of Medicine, especially Dr. Hua Tan, for valuable discussions and advices. This work was supported by National Institutes of Health [1U01CA166886, 1U01AR069395 and 1R01GM123037] (to X. Zhou), and partially supported by NSFC No.61373105 and No.61672422. Funding for open access charge: National Institutes of Health [1U01CA166886]. The authors acknowledge the Texas Advanced Computing Center (TACC) at the University of Texas at Austin (http://www.tacc.utexas.edu < http://www.tacc.utexas.edu/ > ) and the DEMON high performance computing (HPC) cluster at Wake Forest University School of Medicine for providing HPC resources that have contributed to the research results reported within this paper.”

