# Supplementary material for: RPI-Bind: a structure-based method for accurate identification of RNA-protein binding sites
Source: Sci Rep. 2017 Apr 4;7:614. doi: 10.1038/s41598-017-00795-4 (PMC5429624; doi:10.1038/s41598-017-00795-4)
Supplement: Supplementary file 1 — Supplemental Materials [file 41598_2017_795_MOESM1_ESM.pdf]

RPI-Bind: a structure-based method for accurate identification of RNA-protein binding sites

Jiesi Luo[*], Liang Liu[*], Suresh Venkateswaran[*], QianQian Song and Xiaobo Zhou**

Center for Bioinformatics and Systems Biology and Department of Radiology, Wake Forest School of Medicine, Winston-Salem, NC 27157, USA

* Authors contributed equally to this work

** To whom correspondence should be addressed. Tel: +1-336-713-1789; Fax: +1-336-713-5891; Email: xizhou@wakehealth.edu

Key words: protein-RNA binding; binding sites; sequence; structure

Supplemental materials

Section 1. Data set

A total of 1,342 protein-RNA complexes were extracted from the Nucleic Acid Database (NDB)[1] (as of January, 2015), and their corresponding structures, solved by X-rays crystallography with resolution better than 3.5 Å, were downloaded from the Protein Data Bank (PDB)[2]. The polypeptides (protein with sequence length <25 amino acids) and polyribonucleotides (RNA with sequence length <10 nucleotides) were excluded, due to their low information content. Further, to avoid homology bias, a strict criterion was used. Firstly, the non-redundant dataset was obtained by clustering the protein and RNA sequences with CD-HIT program[3] at 40% identity and 80% similarity. Then another common tool, BLASTCLUST program [4] was used to ensure that no clear homologues are present in the non-redundant dataset. In this way, we removed protein and RNA chains with sequence identity above 25% and 60%, respectively. Additionally, similar protein chains interacting with similar RNA chains were considered as homologous RNA-protein pairs. Such pairs were clustered and a representative pair from each cluster was selected. Since the PDB data contained both biological interactions and crystal contacts, it was necessary to distinguish between them because crystal contacts can cause erroneous identification of the interaction. Here, only those RNA-protein pairs containing more than 5 interacting residues/nucleotides were kept as biological interactions. Finally, 172 non-redundant RNA-protein pairs were obtained. (Supplemental Table S1).

Further, we used the following criteria to define protein-RNA contacts: (i) hydrogen bond with maximum donor-acceptor distance <= 3.35Å and maximum hydrogen-acceptor distance <=2.7Å[5,6], and (ii) van der Waals forces with hydrophobic, electrostatic and

distance based interactions <=3.9 Å[7,8]. As a result, we obtained a total of 28,780 contacts, consisting of 9,077 RNA binding sites and 5,692 protein binding sites.

The residues other than binding sites are non-binding sites. However, the non-binding regions between protein and RNAs sometimes is not due to the structures and sequences of RNA or proteins, especially of the local structures of these regions, but the distance from the 3D structures and space. Therefore, the inclusion of all the residues excluding the binding sites may underestimate the roles of structure and sequence in the binding regions. In this work, we mainly focus on the role of local structure and sequence, and we are very curious about whether there are differences between binding sites and their neighbor non-binding sites in the same local structure and sequence environment. So we specifically selected the neighbor non-binding sites as negative samples. Under above criteria, those residues or nucleotides who fail to form hydrogen bond, van der Waals, hydrophobic and electrostatics interactions are defined as non-binding sites. Then a five-length sliding window (the center of each window is binding site) was used to search both sides of binding sites. Those non-binding sites in the window are "neighbor non-binding sites". In this way, we collected 9,801 RNA non-binding sites and 3,078 protein non-binding sites.

Section 2. Structure representations

In this study, protein blocks (PBs) was used to represent protein local conformations (PLCs). PBs is one of the structural alphabets used to describe each and every region of protein backbones. PBs has 16 structural fragments, with each containing 5 residues in length and corresponds to eight dihedral angles (φ,ψ). The structural differences between

PLCs depend on the side chain length, position, and proximity of the dihedral angle to the protein backbone. Each PLC has a unique side-chain conformation; therefore the PLCs can represent the protein side-chains. We used the PDB-2-PB[9] to retrieve the PBs for each protein in our data set (Supplemental Table S2).

The RNA structures can also be approximated by using different approaches[10-14]. In this work, we used the 'BEAR' approach [10] to extract 12 types of RNA local conformations (RLCs) from the corresponding 3D structures. The 12 types of RLCs are Loop, Stem, Stem_branch, Left_internal_loop, Bulge_left, Left_internal_loop_branch, Bulge_left_branch, Right_internal_loop, Bulge_right, Right_internal_loop_branch, Bulge_right_branch and other. These RLCs result in distinct and reproducible backbone conformations therefore; they can represent the RNA backbones. We labeled by alphabetical letters from *a* to *l*, respectively (Supplemental Table S3).

The PLCs and RLCs are similar to the protein- and RNA-secondary structures but the difference is that they contain more structural states than regular secondary structures. So the PLCs and RLCs can be used to predict the potential binding sites not only on protein and RNA bound conformations but also their unbound conformations.

PLCs and RLCs binding preferences

PLCs and RLCs interface preferences were calculated for the non-redundant dataset of protein-RNA complexes. These preferences give a measurement of the possibility of different PLCs and RLCs in the RNA-protein binding interfaces. Taken the PLCs as an example, the interface preferences were calculated as: $P_i^{PLC} = \log_2(f_i^{PLC,binding} / f_i^{PLC,non-binding})$ (*i*=1, 2,…,16), where $f_i^{PLC,binding}$ and

$f_i^{PLC,non-binding}$ are the frequency of i-th PLC type in the RNA binding sites and non-binding sites, respectively. $f_i^{PLC,binding} = N_i^{PLC,binding} / \sum_{i=1}^{16} N_i^{PLC,binding}$, where $N_i^{PLC,binding}$ is the observed number of the i-th PLC type in the RNA binding sites. Same calculations were performed to RLC.

Structures features of proteins and RNAs

In this study, the interacting RNA-protein interacting pairs were measured by using the interaction propensity with log-odds values. Here we considered the structure preference between every three continuous amino acids (triplet) and one contacted RNA nucleotide. We adopted this interaction propensity measurement, because neighboring PLCs/RLCs play an important role in determining the RPI.

The triplet interaction propensities $\mathrm{S(i,j)}$ for all four possible combinations were calculated using following formula: $\mathrm{S(i,j)} = \sum_{\mathrm{(p,r)}} \mathrm{f_{p,r}(i,j)} * \log_2(\frac{\mathrm{f_{p,r}(i,j)}}{\mathrm{f_p(i)} * \mathrm{f_r(j)}})$, where i represents residue triplet, y represents four nucleotides. The $\mathrm{f_{p,r}(i,j)}$ representing frequency of i interacting j in the protein/RNA pair (p, r) was calculated as $\mathrm{f_{p,r}(i,j)} = \mathrm{N_{p,r}(i,j)} / \sum_{\mathrm{i,j}} \mathrm{N_{p,r}(i,j)}$, where $\mathrm{N_{p,r}(i,j)}$ is the observed number of residue triplet i binding to nucleotide j and $\sum_{\mathrm{i,j}} \mathrm{N_{p,r}(i,j)}$is the total number of residue triplets and nucleotides pairs in the given RPI pair (p,r). The $\mathrm{f_p(i)} = \mathrm{N_p(i)} / \sum_{\mathrm{i}} \mathrm{N_p(i)}$ represents the frequency of the residue triplet i in protein p, where $\mathrm{N_p(i)}$ is the number of residue triplet x and $\sum_{\mathrm{i}} \mathrm{N_p(i)}$ is the total number of all residue triplets in the protein p. Similarly, $\mathrm{f_r(j)} = \mathrm{N_r(j)} / \sum_{\mathrm{j}} \mathrm{N_r(j)}$ represents the frequency of a nucleotide j, where $\mathrm{N_r(j)}$ is the number of nucleotides j and $\sum_{\mathrm{j}} \mathrm{N_r(j)}$ is the total number of nucleotides in the RNA r. The triplet log-

odds value of a triplet *i* and a nucleotide *j* were calculated for all interacting protein/RNA pairs in the dataset.

We calculated four types of triplet log-odds values from the RPI sequence and structures as following: (i) amino acid triplets with nucleotides, (ii) nucleotide triplets with amino acids, (iii) PLC triplets with RLCs and (iv) RLC triplets with PLCs. In this work, we used all these four combinations of triplet log-odds value matrices for binding site predictions.

Section 3. Sequence features of proteins

Each amino acid residue is represented by six descriptors including sequence mutual interaction propensities, physicochemical characteristics, hydrophobic index, relative accessible surface area, conservation score and side-chain pKa values, as follows:

The sequence mutual interaction propensities were calculated as triplet-log-odds values[15], similar to the method described in the "Structures features of proteins and RNAs" section.

The physicochemical characteristics of an amino acid residue are described by three values: number of atoms, number of potential hydrogen bonds and number of electrostatic charge[16].

The hydrophobicity is described by the hydrophobic index designed in Sweet and Eisenberg [17]. Relative accessible surface area was calculated by the PSAIA program[18].

The side-chain pKa values, representing protein side-chain environmental properties, were obtained from Wang and Brown[19].

The Position-Specific Scoring Matrix (PSSM) profiles contain the evolutionary information of a protein. Each element in PSSM indicates the probability of the individual residue at that specific position in the multiple sequence alignment[4]. In this

study, the PSSM profile of each sequence was generated by running PSI-BLAST against the Swiss-Prot database with three iterations and a cutoff E-value of 0.001. The generated PSSM profile includes L× 20 elements, where L is the length of protein. We generated a 20-D vector where each element represents the average score of the amino acid residues in a protein being mutated of corresponding amino acid type during the evolution process.

Section 4. Sequence features of RNAs

Each nucleotide in step 2 is also represented by three sequence features: mono-, di- and tri-nucleotide composition. In mono-nucleotide composition, we calculated four nucleotide (A, C, G, and U) compositions in each window sequence separately. In di-nucleotide composition, the composition of two continuous nucleotides (AA, AC, AG, AU ...) in each window sequence was calculated separately, and provided a total of 16 numerical values. In tri-nucleotide composition, we calculated the composition of three continuous nucleotides (AAA, AAC, AAG …) in each window sequence separately.

Section 5. Machine learning methods

The Random Forest (RF) approach is a popular machine learning technique used for dealing with various biological problems[20]. Here, we applied RF classifier implemented by the RF package in *R*, to perform binding site prediction for a given protein chain, RNA chain and protein-RNA pair. The two parameters, *ntrss* (the number of to grow) and *mtry* (the number of variables randomly selected as candidates at each node), were optimized using a grid search approach; the value of *ntrss* was from 500 to 2500 with a step length of 500, and the value of *mtry* was from 1 to 40 with a step length of 1. In addition, the RF provides an important application of selecting more important features based on their contributions to the performance of the predictive models. Permutation

importance analysis is frequently used as a metric in the RF method for measuring the relative importance of features, and the importance score of a feature is calculated according to the average decrease of the model accuracy on the out-of-bag samples when this feature is randomly permuted. Here, the RF and permutation importance analysis were implemented by the RF package in R.

We also compared the performance of the RF method with other machine learning methods, including Support Vector Machine (SVM) and Neural Network (NN). For SVM, we considered the radial basis function (RBF) as the kernel function, and two parameters, the regularization parameter C and the kernel width parameter γ were optimized by using a grid search approach. It could identify good parameters based on exponentially growing sequences of (C, γ) (for example, C $=2^{-5}$, 2^{-4}… 2^{10} and γ = 2^{-10}, 2^{-9}… 2^{5}). A standard feed-forward neural network was used, with a sigmoid transfer function and a single hidden layer of 10 neurons. All possible connections were allowed between the input units and the hidden-layer neurons, as well as between the former neurons and the final output units. The backpropagation algorithm was applied in training the ANN, with random initial weights. The learn rate was set to 0.0001 and the weight decay to -0.001. SVM and NN were implemented in the python module scikit-learn.

Section 6. Performance evaluation

Five-fold cross validation was used to evaluate the performance of our models. The data set was divided into 5 subsets of equal size. Each subset was used for testing, while the remaining four subsets were used for training. This process was repeated 5 times to cover all possibilities. Using the numbers of true positives (TP), true negatives (TN), false positives (FP) and false negatives (FN), four measures Sensitivity (SN), Specificity (SP),

Accuracy (ACC) and Matthew's Correlation Coefficient (MCC) were calculated to determine the prediction performance:

$$ACC = \frac{(TP + TN)}{(TP + TN + FP + FN)}$$

$$SN = \frac{TP}{(TP+FN)}$$

$$SP = \frac{TN}{(TN+FP)}$$

$$MCC = \frac{TP \times TN - FP \times FN}{\sqrt{(TP + FN) \times (TP + FP) \times (TN + FP) \times (TN + FN)}}$$

In addition, receiver-operating characteristic (ROC) curve and the area under ROC curve (AUC) were also used to visualize and evaluate the prediction performance.

Reference

1 Coimbatore Narayanan, B. *et al.* The Nucleic Acid Database: new features and capabilities. *Nucleic acids research* **42**, D114-122, doi:10.1093/nar/gkt980 (2014).

2 Rose, P. W. *et al.* The RCSB Protein Data Bank: redesigned web site and web services. *Nucleic acids research* **39**, D392-401, doi:10.1093/nar/gkq1021 (2011).

3 Li, W. & Godzik, A. Cd-hit: a fast program for clustering and comparing large sets of protein or nucleotide sequences. *Bioinformatics* **22**, 1658-1659, doi:10.1093/bioinformatics/btl158 (2006).

4 Altschul, S. F. *et al.* Gapped BLAST and PSI-BLAST: a new generation of protein database search programs. *Nucleic Acids Res* **25**, 3389-3402, doi:DOI 10.1093/nar/25.17.3389 (1997).

5 Jones, S., Daley, D. T. A., Luscombe, N. M., Berman, H. M. & Thornton, J. M. Protein-RNA interactions: a structural analysis. *Nucleic Acids Res* **29**, 943-954, doi:Doi 10.1093/Nar/29.4.943 (2001).

6 Ellis, J. J., Broom, M. & Jones, S. Protein-RNA interactions: structural analysis and functional classes. *Proteins* **66**, 903-911, doi:10.1002/prot.21211 (2007).

7 Ellis, J. J. & Jones, S. Evaluating conformational changes in protein structures binding RNA. *Proteins* **70**, 1518-1526, doi:10.1002/prot.21647 (2008).

8 Murakami, Y., Spriggs, R. V., Nakamura, H. & Jones, S. PiRaNhA: a server for the computational prediction of RNA-binding residues in protein sequences. *Nucleic Acids Res* **38**, W412-W416, doi:10.1093/nar/gkq474 (2010).

9 Suresh, V., Ganesan, K. & Parthasarathy, S. PDB-2-PB: a curated online protein block sequence database. *J Appl Crystallogr* **45**, 127-129, doi:10.1107/S0021889811052356 (2012).

10 Mattei, E., Ausiello, G., Ferre, F. & Helmer-Citterich, M. A novel approach to represent and compare RNA secondary structures. *Nucleic acids research* **42**, 6146-6157, doi:10.1093/nar/gku283 (2014).

11 Zheng, G. H., Lu, X. J. & Olson, W. K. Web 3DNA-a web server for the analysis, reconstruction, and visualization of three-dimensional nucleic-acid structures. *Nucleic Acids Res* **37**, W240-W246, doi:10.1093/nar/gkp358 (2009).

12 Liu, L. A. & Chen, S. J. Computing the conformational entropy for RNA folds. *J Chem Phys* **132**, doi:Artn 23510410.1063/1.3447385 (2010).

13 Liu, L. & Chen, S. J. Coarse-Grained Prediction of RNA Loop Structures. *Plos One* **7**, doi:ARTN e4846010.1371/journal.pone.0048460 (2012).

14 Chen, S. J. RNA folding: Conformational statistics, folding kinetics, and ion electrostatics. *Annu Rev Biophys* **37**, 197-214, doi:10.1146/annurev.biophys.37.032807.125957 (2008).

15 Liu, Z. P., Wu, L. Y., Wang, Y., Zhang, X. S. & Chen, L. N. Prediction of protein-RNA binding sites by a random forest method with combined features. *Bioinformatics* **26**, 1616-1622, doi:10.1093/bioinformatics/btq253 (2010).

16 Li, N., Sun, Z. H. & Jiang, F. Prediction of protein-protein binding site by using core interface residue and support vector machine. *Bmc Bioinformatics* **9**, doi:Artn 55310.1186/1471-2105-9-553 (2008).

17 Sweet, R. M. & Eisenberg, D. Correlation of Sequence Hydrophobicities Measures Similarity in 3-Dimensional Protein-Structure. *Journal of molecular biology* **171**, 479-488, doi:Doi 10.1016/0022-2836(83)90041-4 (1983).

18 Mihel, J., Sikic, M., Tomic, S., Jeren, B. & Vlahovicek, K. PSAIA - Protein structure and interaction analyzer. *Bmc Struct Biol* **8**, doi:Artn 2110.1186/1472-6807-8-21 (2008).

19 Wang, L. J. & Brown, S. J. BindN: a web-based tool for efficient prediction of DNA and RNA binding sites in amino acid sequences. *Nucleic Acids Res* **34**, W243-W248, doi:10.1093/nar/gkl298 (2006).

20 Breiman, L. Random forests. *Mach Learn* **45**, 5-32, doi:Doi 10.1023/A:1010933404324 (2001).

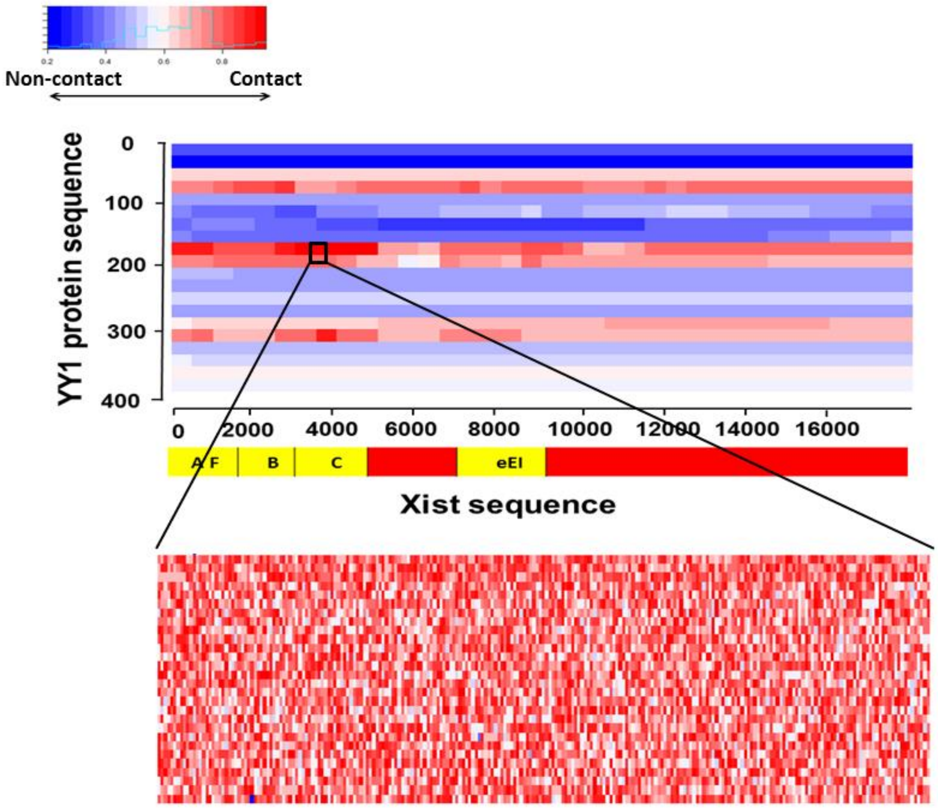

Supplemental Figure S1. (A) The prediction of binding interactions between *Xist* and transcriptional repressor YY1. (B) The detail prediction of binding interactions between the YY1 segment (sequence from 170-200) and *Xist* segment (sequence from 2500-2800).

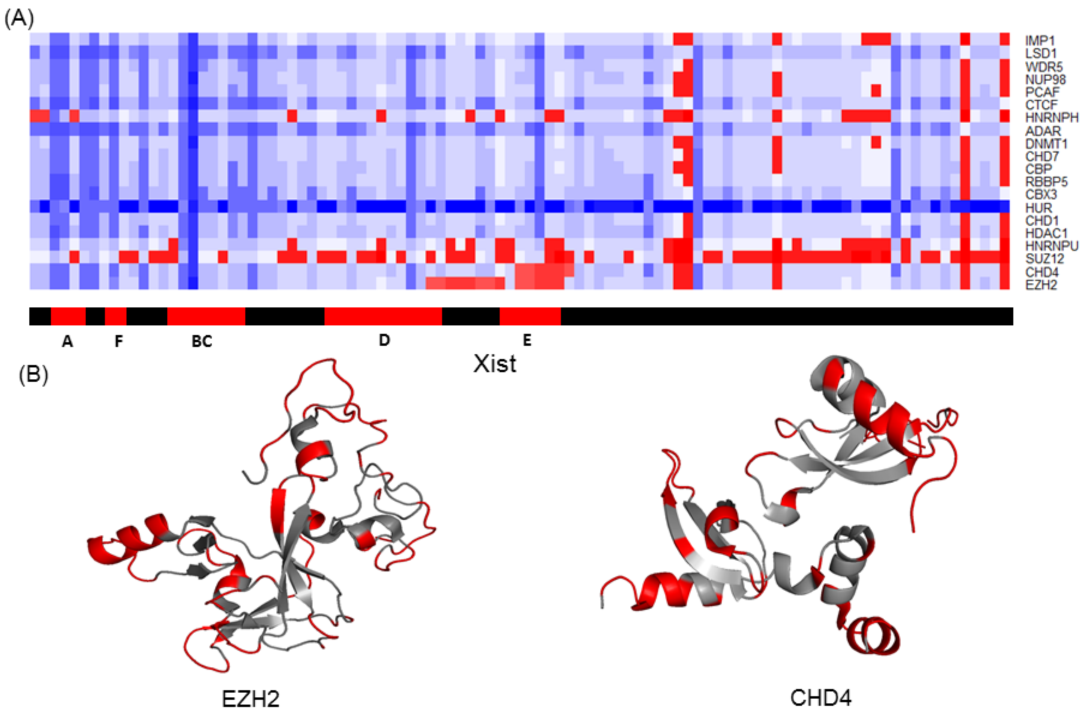

Supplemental Figure S2. Chromatin-associated proteins and lncRNA *Xist* interactions. (A) The heat map shows the prediction results of *Xist* binding by 20 proteins. The results are color-coded: red represents high probability contacts and blue represents non-contacts. (B) Examples of predicted *Xist*-protein interacting complexes. Two proteins are EZH2 and CHD4. The results are mapped onto the original structure where different prediction scores are represented by different colors.

Supplemental Tables

Supplemental Table S1. 172 non-redundant RNA-protein pairs

Supplemental Table S2. The 16 types of protein local conformations (PLCs), including labels, names of local conformations, occurrences of PLC at and outside the interface for all four classes (enzymes, structural, regulatory and other).

Supplemental Table S3. The 23 types of RNA local conformations (RLCs), including label, name of local conformations, occurrences of RLCs at and outside the interface for all four classes (enzymes, structural, regulatory and other).

Supplemental Table S4. The prediction accuracy for each protein-RNA complex
